# North-West London Hospital

**Published:** 1902-04-12

**Authors:** 


### NORTH-WEST LONDON HOSPITAL.

### NO IMMEDIATE PROSPECTS FOR THE NEEDFUL NEW BUILDINGS.

The annual general court of governors and subscribers of the North-West London Hospital was held at the hospital on Monday. Lord Rathmore presided, and the attendance —possibly owing to the weather prevailing—was very small.

The Secretary (Mr. Alfred Craske) read the report of the committee, which states that they are convinced that the record of the institution's work not only emphasises the great need there is for the hospital, but also demonstrates the inadequacy of the accommodation provided by the present building and premises for the ever-increasing demands upon the resources of the institution. During the year 639 in-patients and 19,556 out-patients, including 6,120 casualties, were treated. The total number of attendances of out-patients was 44,246. In accordance with the wish of the council of King Edward's Fund the committee are about to provide new bath-room accommodation. The total income for the year amounted to £3,166, which includes: Donations, £1,092; annual subscriptions, £621; and legacies, £225. The expenditure was £4,850—an excess over income of £1,684. During the year a special committee was formed to deal with the question of providing a new building for the hospital. The difficulty, however, of raising funds for meeting current expenses has been an insuperable obstacle in the way of proposing any definite mode of action to accomplish this object. The committee record their grateful appreciation of Mr. George Herring's generosity in maintaining the soup kitchen close to the hospital, from which the poorer out-patients have derived much benefit; and their cordial thanks for the various contributions and gifts, and the services of the honorary medical staff. The yearly decreasing income from annual subscriptions is much to be regretted. The committee point out the urgent need of a largely increased revenue from this source, which should be made the mainstay of the hospital for satisfactorily carrying on its work.

The Chairman, in moving the adoption of the report, said their accounts showed that the utmost care and economy were exercised in the application of funds placed at their disposal. No one could read the report without mingled feelings, for it was very regrettable that their subscriptions and income did not keep pace with the expenditure. Unfortunately, this state of affairs precluded the committee going on, for the time being, with the scheme for the provision of a better building in which to continue the work of the hospital; but it was plain that if they could not obtain sufficient funds to meet their expenditure it would be almost impossible to succeed in obtaining such a large sum of money as was necessary to acquire a new building. However, there was a good deal in the committee's report from which those who subscribed and those who gave their services to the hospital might derive much satisfaction. The number of out-patients had proved the necessity of such an institution as that in the neighbourhood. The number of patients treated spoke eloquently of the great amount of relief which must have been given during the year. That the quality of the work done in the hospital was of the first kind was demonstrated by the fact that they had again received from King Edward's Hospital Fund for London a donation of £500, which was made more gratifying by the message of approval which accompanied it. The message became even more valuable when they remembered that no donation was ever made from the King's Hospital Fund until an inspection of the hospital had been made by independent medical experts. Having acknowledged the kindness of Mr. George Herring and the contributors and the services of the medical and nursing staffs, Lord Rathmore reverted to the financial condition of affairs. He said the appeal they made met with very unsatisfactory response. This was not a good time for such an appeal, and they must look forward to a better condition of affairs when he hoped they would meet with more success. In the meantime they must rely on their old friends to continue their subscriptions and if possible increase them. It was obvious that unless the subscriptions increased it would be impossible to continue the services which the hospital rendered.

Mr. Bangs seconded the motion, which was carried.

The committee of management were re-elected, and the medical and nursing staffs were cordially thanked for their services. A vote of thanks to the chairman concluded the meeting.

